# Cancer microarray data feature selection using multi-objective binary particle swarm optimization algorithm 

**DOI:** 10.17179/excli2016-481

**Published:** 2016-08-01

**Authors:** Chandra Sekhara Rao Annavarapu, Suresh Dara, Haider Banka

**Affiliations:** 1 Department of Computer Science and Engineering, Indian School of Mines, Dhanbad-826004, Jharkhand, India

**Keywords:** cancer micro array, gene expressions, feature selection, binary PSO, classification

## Abstract

Cancer investigations in microarray data play a major role in cancer analysis and the treatment. Cancer microarray data consists of complex gene expressed patterns of cancer. In this article, a Multi-Objective Binary Particle Swarm Optimization (MOBPSO) algorithm is proposed for analyzing cancer gene expression data. Due to its high dimensionality, a fast heuristic based pre-processing technique is employed to reduce some of the crude domain features from the initial feature set. Since these pre-processed and reduced features are still high dimensional, the proposed MOBPSO algorithm is used for finding further feature subsets. The objective functions are suitably modeled by optimizing two conflicting objectives i.e., cardinality of feature subsets and distinctive capability of those selected subsets. As these two objective functions are conflicting in nature, they are more suitable for multi-objective modeling. The experiments are carried out on benchmark gene expression datasets, i.e., Colon, Lymphoma and Leukaemia available in literature. The performance of the selected feature subsets with their classification accuracy and validated using 10 fold cross validation techniques. A detailed comparative study is also made to show the betterment or competitiveness of the proposed algorithm.

## Introduction

Cancer treatments are targeted for therapies to distinct tumour types by using many computational methods to analyze cancer data, cancer deaths are more than heart disease in persons younger than 85 years (Jemal et al., 2010[14]). Cancer tissue classification is used for diagnosing the cancer. Cancer classification based on gene expression monitoring is used to discover and predict cancer classes of all types without prior biological knowledge (Golub et al., 1999[7]). Prior to classification, finding relevant genes are highly significant to classifying the cancer microarray data. Only few relevant genes are important in the classification. Irrelevant genes cause for low accuracy in classification by hiding relevant features (Guyon et al., 2002[8]). It is therefore not surprising that much effort have been put into developing methods for gene selection (Saeys et al., 2007[27]).

Microarray data, involves the decoding of approximately 30000 human genes, a kind of NP-Hard problem (Banerjee et al., 2007[1]). Feature selection technique on high dimensional helps to identify key features, also reduces the computational cost and increases the classifier performance. For classifier accuracy in DNA microarray many methods have been proposed, ONCOMINE platform, which is a collection of many gene expression dataset for enlarging its research (Rhodes et al, 2004[26]), recent studies done by (Hatzimichael et al, 2014[10]; Lu et al., 2014[21]) reveals its demand, clustering (Mitra and Ghosh, 2012[23]), and feature selection (Lazar et al., 2012[17]; Linde et al, 2015[20]; Kurakula et al, 2015[16]; Marchan, 2015[22]; Chandrashekar and Sahin, 2014[2]) are recent trends in the research. Hence, expression profiling or microarray gene expression data analyses are prominent tasks in this field.

Feature selection methods selects a subset of 'd' features from a set of 'n' features on the basis of optimization methods. There are many high dimensional datasets which have thousands of features and many of them are irrelevant or redundant. Unnecessary features increase computational burden and make generalization more difficult (Lazar et al., 2012[17]). The feature selection techniques are important tool to reduce dimensionality and to select useful feature subsets that maximizes the classification accuracy (Saeys et al., 2007[27]).

Feature selection methods can be categorized as: filter based, wrapper based, embedded/hybrid based and ensemble methods (Lazar et al., 2012[17]). Filter techniques (Elalami, 2009[6]), selects feature subsets independently of any learning algorithm, assess a significant score with a threshold value to choose the best features. The wrapper model (Sainin and Alfred, 2011[28]) uses predictive accuracy of predetermined learning algorithms. The embedded techniques (Wahid et al, 2011[32]) allow interaction of different class of learning algorithms. More recently, the ensemble model (Nagi and Bhattacharyya, 2013[25]) based on different sub sampling strategies, the learning algorithms run on a number of sub samples and the acquired features are united into a stable subset. However the feature selection techniques can be also categorized based on search strategies used such as forward selection, backward elimination, forward stepwise selection, backward stepwise selection and random mutation (Mladeni, 2006[24]).

Feature selection algorithms are to find feature subsets which are validated by classification accuracy for checking its performance (Yu et al., 2008[33]).

**Figure 1:**
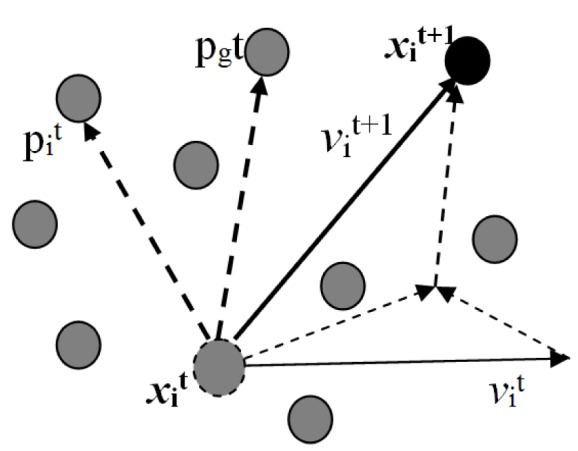
An illustration of PSO architecture

1. Evolutionary computation is a biologically inspired meta-heuristic used for search and optimization representing a powerful and rapidly growing field of artificial intelligence. It uses natural genetics and natural selection to evolve a population of candidate solutions for a given problem. In this paper, we presented a multi objective BPSO algorithm to select feature subsets from high dimensional gene expression data. PSO (see Figure 1(Fig. 1)) has certain merits with respect to others such as: i) it uses less number of parameters;
2. it may converge faster and has less computational burden and
3. having potential accuracy.

We proposed a BPSO that preserves better solutions for the next generation. At the first stage, the data is normalized, discretized and converted to binary distinction table by reducing the dimensionality of each sample. At the second stage, BPSO is used to select the significant feature subsets. External validation of selected feature subsets is done in terms of classification accuracy with standard classifiers (Hall et al., 2009[9]).

The remaining part of the paper is structured as follow. The second section describes the preliminaries of PSO algorithm and dominance criteria. The third section discusses about pre-processing of gene expression data, objective functions and the proposed MOBPSO. The fourth section is about various results on three cancerous microarray data such as colon, lymphoma and leukaemia with their validation through standard machine learning classifiers. The last section concludes the article.

## Preliminaries

This section formally describes the basics of micro array gene expression data, binary particle swarm optimization algorithm, the dominance criteria with non-dominated sorting algorithm that are relevant for understanding the present work. 

### Micro array gene expression data

In early 1980's the Array technology was started, did not come into prominence until the mid-1990. But, with the introduction of cDNA microarray technology got lot of fame (Sun et al., 2013[31]). Today, microarrays researchers using array technology in genomic research with a diversified range of applications in biology and medicine. A few recent applications include microbe identification, tumour classification, evaluation of the host cell response to pathogens and analysis of the endocrine system (Konishi et al., 2016[15]).

Analysing DNA microarray data requires a pre-processing phase to produce new biological assumptions, this phase involves distribution, normalization and gene filtering and discretization (Lévêque et al., 2013[18]).

Microarray data classification, which predicts the diagnostic category of a sample from the expression array is a kind of supervised learning. Microarray with orderly arranged samples, provides a good media for matching known and unknown DNA segments with the help of base pairing rules. Microarrays produces huge information requires a series of repeated analyses to render the data interpretable and find out hidden information or pattern in them. The direct output of microarrays is difficult to distinguish various conditions of the samples, or the time points.

### Multi objective optimization

Multi objective optimization involves more than one objective function to get optimal solutions. This involves optimization of single objective function with a trade-off between different objectives, multi objective optimization is also achieved through Particle Swarm Optimization (Coello and Lechuga, 2012[4]).

### BPSO (binary particle swarm optimization)

Particle swarm optimization is a heuristic, multi-agent, optimization and evolutionary technique (James and Russell, 1995[13]). It is found to be robust in solving problems featuring nonlinearity, non-differentiability, multi criteria, and high-dimensionality through adaptation which is derived from social-psychological theory (James and Russell, 1997[12]). 

The progress of every particle is calculated as per the defined fitness function (James, 1997[11]) and is updated in their velocities and positions according to the following equation.


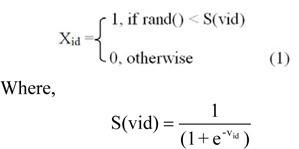


Where rand() is a function, to generate a uniform distributed random number in [0,1].

### Dominance criteria and non-dominated sorting

In dominance criteria, concept of optimality lies among set of solutions. Solution is said to be dominated with respect to the other solutions based on certain conditions. Non-dominated set in a population are identified with the non-dominated sorting algorithm described in (Deb, 2001[5]).

In this paper, we used two objective functions for finding the non-dominated set among the populations and then assigned the ranks accordingly.

## Proposed Methodologies

This section describes the basic pre-processing of gene expression data, objective functions formulation and its justification, followed by the proposed MOBPSO algorithm.

### Pre-processing gene expression data

Pre-processing aims to eliminate the ambiguously expressed genes. During feature subset generation, appropriate smallest set of differentially expressed genes are selected across the classes for efficient classification.

**Figure 2:**
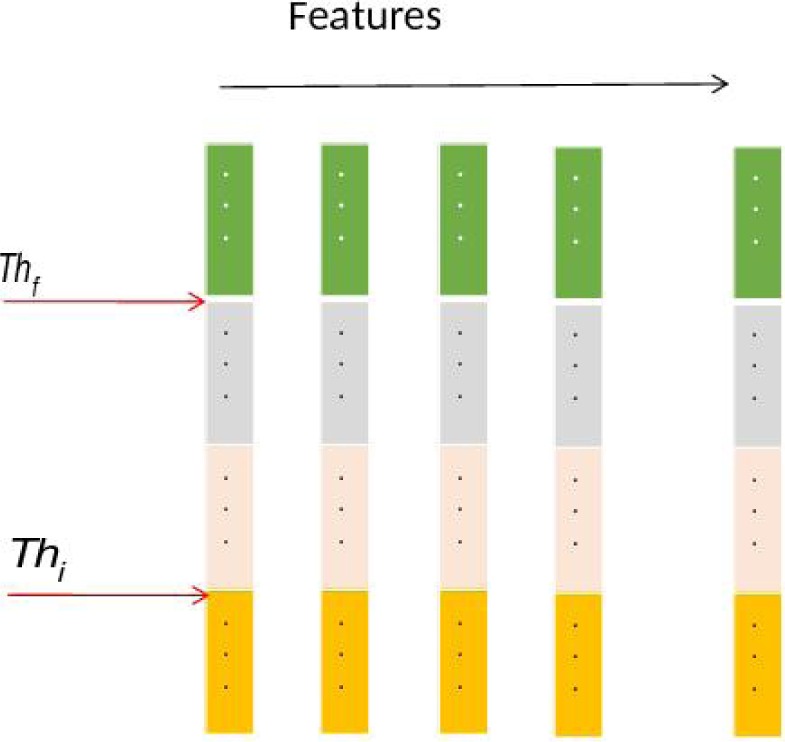
Quartile graph

1. The normalization is to make the values lie between 0.0 to 1.0. Attribute wise normalization is done by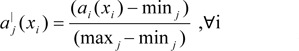  where max_j_ maximum and min_j_ minimum to the gene expression values for attribute a_j_ over all samples. This makes the normalized continuous attribute value in the range 0 to 1.
2. Then two thresholds Th_i_ and Th_f_, based on the idea of quartiles, are chosen, as in (Banerjee et al., 2007[1]). Let N be the number of patterns in the dataset. The measurements are divided into a number of small class intervals of equal width δ and the corresponding count of class frequencies are fr_c_. The position of the k^th^ partition value (k = 1,2,3 for four partitions) is calculated as
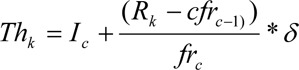
  where l_c_ is the lower limit of the c^th^ class interval,
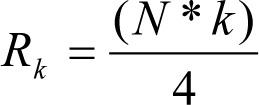
  is the rank of the k^th^ partition value, and cfr_c-1_ is the cumulative frequency of the preceding class such that cfr_c-1_ ≤ R_k_ ≤ cfr_c_. This has been sketched in the following Figure 2(Fig. 2).
3. Converting the attribute value table to binary (0/1) form as follows:
4. If a*^|^*(x) ≤ Th_i_ Then put '0', Else If a*^|^*(x) ≥ Th_f_ Then put '1', Else put '*' (don't care).
5. Find the average occurrences of '*' as threshold Th_a_.
6. The attributes whose number of '*'s are ≥ Th_a_ are removed from the table This is the modified (reduced) attribute value table F_r_.

After this, the number of features in distinction table becomes 1102 features from 2000 features for colon, become 1867 features from 4026 for lymphoma, and become 3783 features from 7129 for leukemia dataset.

### Distinction table preparation

To make a distinction table, a matrix of binary values with dimensions


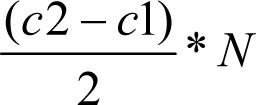


is defined, where N is the number of features in F, C is the number of objects/samples. An entry b((k,j),i) of the matrix corresponds to pair of objects (x_k_,x_j_) and with the attribute a_i_.


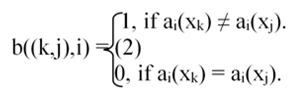


The presence of a '1' signifies the attribute a_i_'s ability to distinguish (or discern) between the pair of objects (x_k_, xj).

For a decision table F with N condition attributes and a single decision attribute d, the problem of finding a reduct is equivalent to finding a minimal subset of columns R(⊆ {1,2,··· ,N}) in the distinction table using (4), satisfying ∀(k,j)∃i ∈ R : b((k,j),i) = 1,whenever d(x_k_) ≠ d(x_j_). 

So, in effect, we may consider the distinction table to consist of N columns, and rows corresponding to only those object pairs (x_k_,x_j_) such that d(x_k_) ≠d(x_j_).

- As object pairs corresponding to the same class do not constitute a row of the distinction table, there is a considerable reduction in its size thereby leading to a decrease in computational cost.
- Additionally, If either of the objects in a pair, has '∗' as an entry under an attribute in table F_r_ Then in the distinction table, put '0' at the entry for that attribute and pair.
- The entries '1' in the matrix correspond to the attributes of interest for arriving at a classification decision.

If C_1_ and C_2_ are the number of objects of the two classes respectively, then rows of the distinction table turn out to be


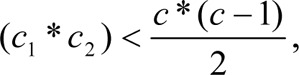


where C_1_+C_2_ = C. This reduces time complexity of fitness computation to O(N ∗ C_1_ ∗ C_2_).

Table 1(Tab. 1) describes how a sample distinction table looks like. Here, assume that there is seven conditional features {F_1_, F_2_, F_3_, F_4_, F_5_, F_6_, F_7_}, the length of vector is N = 7. In a vector v, the binary data '1' represents if the corresponding feature is 'present', and a '0' represents its absence. The two classes are C_1_ (with two objects i.e. C_11_ and C_12_) and C_2_ (with three objects i.e. C_21_, C_22_ and C_23_). The rows represent the object pairs and columns represent the features or attributes. The objective is to choose minimal number of column (features) from the table that covers all the rows (i.e., object pairs in the table). Note that, for multi class problem, if there are k number of classes in a particular dataset, there will be ^k^C_2_ number of rows in the distinction table. Therefore, the proposed method is not only limited to solve two class problems, but multi-class problem also. However, the present work is focused on two class problems for benchmark datasets as available in literature.

### Objective functions design

We used two objective functions Fit_1_ and Fit_2_. Objective Function 1: The first objective function F_1_ is used to finds number of features (i.e. number of 1's). The proposed first objective function is as follow:


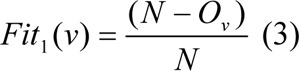


Objective Function 2: The second objective function F_2_ decides the extent to which the feature can recognise among the objects pairs.


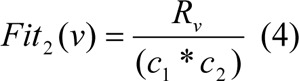


Here, v is the chosen feature subsets, O_v_ represents the number of 1's in v, C_1_ and C_2_ are the number of objects in each of the class and R_v_ is the number of object pairs (i.e. rows in the distinction table) v can discern between. The objective function Fit_1_ gives the candidate credit for containing less number of features or attributes in v, and Fit_2_ determines the extent to which the candidates can discern among objects pairs in the distinction table. 

In simple GA, the two objective functions are combined into one by weighted sum as Fit = Fit_1_ ∗ α + Fit_2_ ∗(1−α), where 0 < α_1_ < 1. 

As an example to calculate Fit_1_ and Fit_2_, let us take a sample input vector v = (1,0,1,1,0,1,1), Two classes are C_1_ and C_2_, where class lengths are C_1_ = 2, C_2_ = 3, and length of vector is N = 7 (as depicted in table 1(Tab. 1)). The number of 1's in v is O_v_ = 5, and R_v_ is calculated as compare with input vector v matching number of presented 1's from each row in distinction table, i.e R_v_ = 5. Therefore


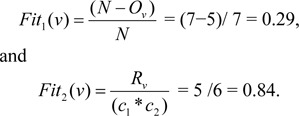


Here, a multi objective BPSO algorithm is proposed for feature subset selection. The best non-dominated solutions of combined population of swarm at two successive generations (i.e., current and next population) are preserved at each generation. Only best 50 % solutions are allowed to evolve for the next generation. This is repeated for finite number of generations. The proposed approach is described in Algorithm [1].

### The MOBPSO algorithm for feature selection


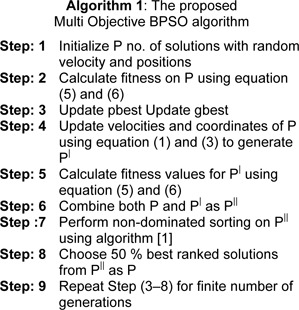


### gbest selection

After perform non-dominating sorting on mixed population (i.e. parent and child), we can get non dominated solutions. Here, we choose one random solution as gbest among top raked non-dominated solutions. Since, more than one top ranked solutions are may be available, but all solutions having same priority.

## Results and Discussions

### Cancer gene expression data sets

In this study, three benchmark cancer datasets have been used for training and testing purpose.

1. Colon Cancer dataset available at (http://genomics-pubs.princeton.edu/ oncology/) is a set of 62 gene expressions, containing 2000 genes (features).
2. Lymphoma dataset available at (http://llmpp.nih.gov/lymphoma/data/figure1/figure1.cdt) is a set of 96 gene expressions, having 4026 genes.
3. Leukaemia dataset available at (http://www.genome.wi.mit.edu/MPR) is a set of 38 gene expressions, having 7129 genes.

Our proposed MBPSO on colon microarray, lymphoma microarray, and leukemia microarray obtained minimal subsets of features. In our experiment values of accelerator coefficients c_1_ and c_2_ are set to 2 whereas velocities set to minimum of -4 and maximum of 4 (Sudholt and Witt, 2008[30]). In BPSO, inertia weight (w) treated as one of the most important parameter, through which we can improve accuracy by estimation and balancing of local and global search (Shi and Eberhart, 1999[29]). After several experiments, 'w' was set to 0.9. Various population sizes were taken, to check feature subsets behaviour, also the swarm size set as per literature. After several experiments maximum number of runs was set to 50 which were also tested with varied population size like 10, 20, 30 and 50. Many standard classifiers have been used for testing purposes to show consistent performance and robustness of the proposed method. The experimental results are carried out on three bench mark datasets as summarized in Table 2(Tab. 2) and 3(Tab. 3).

Note that k is chosen to be an odd number to avoid the ties. The correct classification are reported to be 93.54 %, 95.85 % and 94.74 % for those three datasets with varies swarm size and k values. The results shown above is based on average score over (10- 15) runs. Table 3(Tab. 3) represents k−NN classification results with single objective function of GA. Here, it is giving 100 % correct classification score for all three data sets when k = 1. For colon data 93.55 % score when k = 3, 90.33 % for k = 5 and for k = 7 it is 83.88 %, on 10 feature subset.

For lymphoma data, it is 93.75 %, 93.75 % and 89.59 % where k = 3, k = 5 and k = 7 respectively. Similarly, for leukemia data, where k = 3, 5 and 7 the correct classification is 94.74 %.

Table 4(Tab. 4) depicts the results for the all three datasets using Bayes Family classifiers. The Bayes Logistic Regression (BLR), Bayes Net(BN) classifier, and Naive Bayes(NB) classifiers given 93.55 % highest correct classification result on 13 features subset for colon data. For the lymphoma data, 100 % correct classification score has been achieved by using Bayes Logistic Regression whereas Bayes Net classifier gives 95.84 % and Naive Bayes classifiers gives 97.42 % on 22 feature subset. Similarly for leukemia data, it is 92.1 % classification with Bayes Logistic Regression, 89.48 % with other two classifiers on 14 feature subset.

We investigated different well known function based classifiers such as LibLinear, LibSVM, Logistic, Multilayer perceptron (MLP), stochastic gradient descent (SGD) and Spegasos, reported in Table 5(Tab. 5). For colon data, 100 % correct classification score using all classifiers with 13 and 9 gene subsets except SGD and SPegasos classifiers, where those are giving 96.78 % as highest classification on 13 gene subset. For lymphoma data, same table depicts, 100 % score for all classifiers with various (i.e. 15 to 22) gene subset. For leukemia data, shows that 100 % correct classification score for all classifiers with various gene subsets except SGD and SPegasos classifiers, where those are giving 94.71 % and 93.37 % as highest classification on 13 gene subset.

Table 6(Tab. 6) shows that results of various well known Tree based classifiers such as Best First Decision (BFT), Decision Tree (DT), Functional tree (FT), Decision tree classifier (J48), Logistic model tree (LMT), Randomforest (RF) and reduced error pruning tree (REPT). From the table we observe that, except BFT and DT classifiers, remaining all classifiers are giving 100 % correct classification score at various selected subsets for all three data sets. The BFT giving 93.55 % correct score for colon data, 97.91 % for lymphoma data, and 97.37 % for leukemia data. And the Decision Stump classifiers are giving 83.88 % for colon data with 10 and 6 gene subset, 87.50 % for lymphoma data with 14 gene subset and it is 86.85 % for leukemia data with 11 gene subset. We achieved 100 % correct classification on some not included in the Table 6(Tab. 6) Alternating Decision Tree, Extra Tree, LADTree and Random Tree classifiers. The classifiers are shown in Table 6(Tab. 6).

### K-fold cross validation

Cross-validation techniques are a thorough computational mechanism to estimate performance by the use of examples as training and testing sets. In K-fold cross validation, mimics the training and test sets by repeatedly training the algorithm K times with a fraction 1/K of training examples left out for testing purposes. We use K = 10, which is also called 10-fold cross validation, in each experimental run, nine folds are used for training and remaining one fold is used for testing. Therefore, training and test sets consist of 90 % and 10 % of data (Zhang, 2011[34]). Our 10-fold cross validation result on colon, lymphoma and leukemia datasets reported in Table 7(Tab. 7).

### Comparisons

(Liang et al., 2013[19]) introduces a new evolving personalized modelling method and system (evoPM) that integrates gravitational search inspired algorithm (GSA) for selecting informative features. Here, they used 4 high dimensional benchmark datasets, and reported the selected feature subsets with a minimum of 25 features and maximum of 101 features. In our algorithm, selected feature subsets are 9 to 22. Moreover, their classification accuracy is 87.1 % for colon, 94.81 % for lymphoma, and it was 97.22 % for leukemia data. The proposed MOBPSO performs 100 % classification by some of the classifiers on these datasets. 

**Figure 3:**
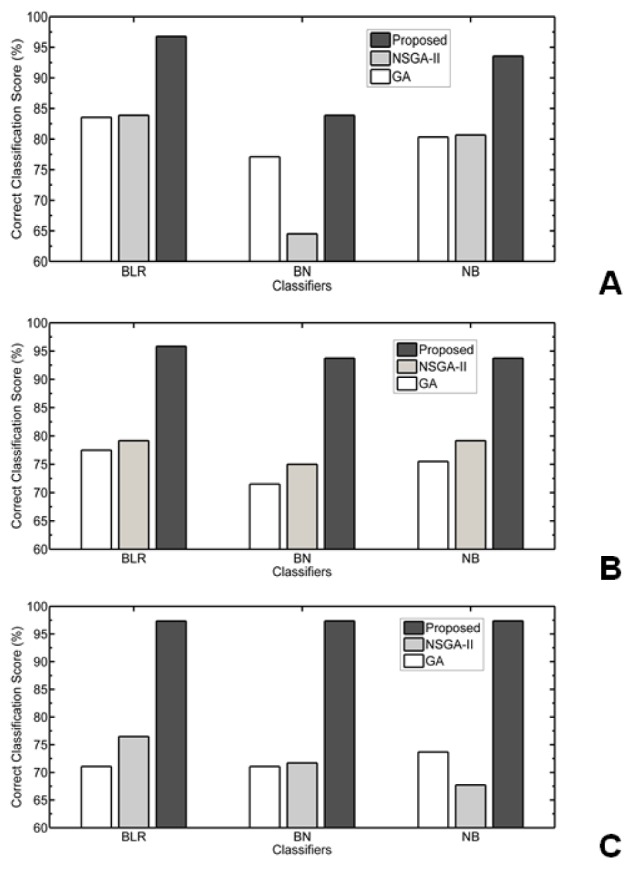
Performance of Proposed MOBPSO Algorithm, NSGA-II and GA on three datasets using bayes Classifiers (BLR, BN, NB); (A) performance of Proposed MOBPSO Algorithm, NSGA-II and GA on Colon dataset using bayes Classifiers (BLR, BN, NB), (B) performance of Proposed MOBPSO Algorithm, NSGA-II and GA on lymphoma dataset using bayes Classifiers (BLR, BN, NB), (C) performance of Proposed MOBPSO Algorithm, NSGA-II and GA on leukemia dataset using bayes Classifiers (BLR, BN, NB)

**Figure 4:**
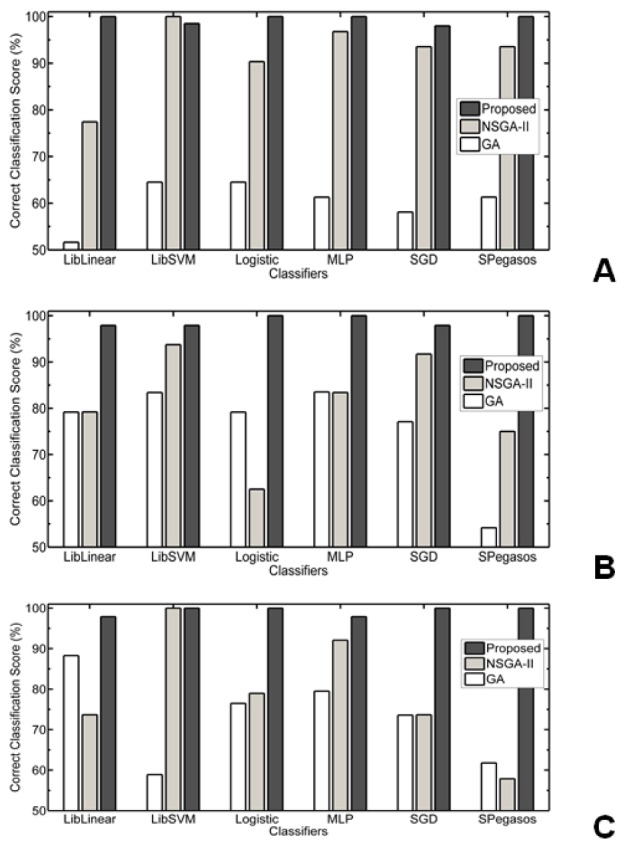
Performance of Proposed MOBPSO Algorithm, NSGA-II and GA on three datasets using Function based Classifiers; (A) performance of Proposed MOBPSO Algorithm, NSGA-II and GA on Colon dataset using Function based Classifiers, (B) performance of Proposed MOBPSO Algorithm, NSGA-II and GA on lymphoma dataset using Function based Classifiers, (C) performance of Proposed MOBPSO Algorithm, NSGA-II and GA on leukemia dataset using Function based Classifiers

**Figure 5:**
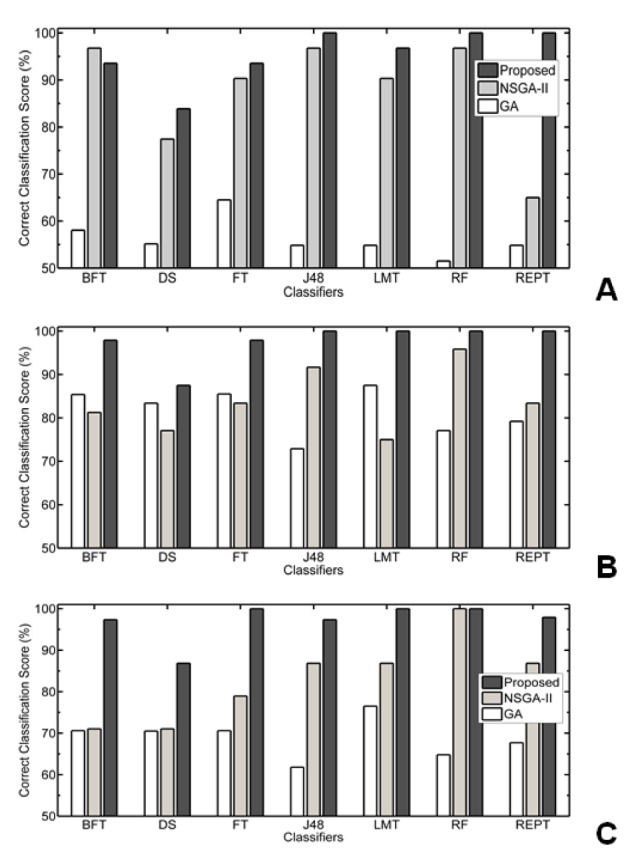
Performance of Proposed MOBPSO Algorithm, NSGA-II and GA on three datasets using Tree based Classifiers; (A) performance of Proposed MOBPSO Algorithm, NSGA-II and GA on Colon dataset using Tree based Classifiers, (B) performance of Proposed MOBPSO Algorithm, NSGA-II and GA on lymphoma dataset using Tree based Classifiers, (C) performance of Proposed MOBPSO Algorithm, NSGA-II and GA on leukemia dataset using Tree based Classifiers

**Figure 6:**
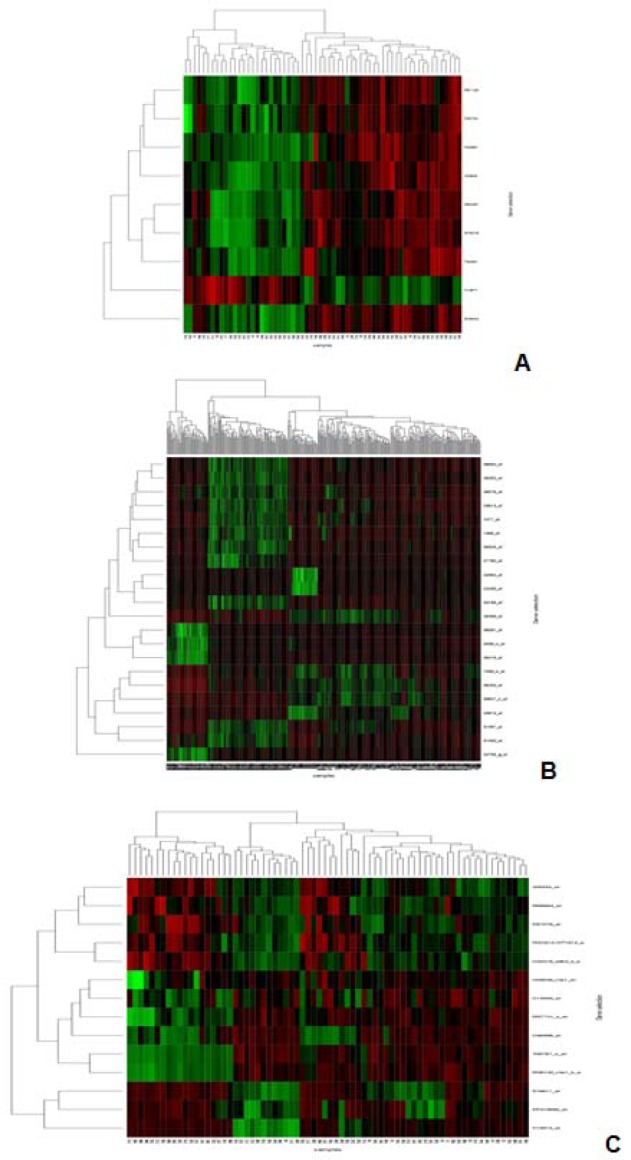
Heat map of three datasets with reduced features; (A) heat map on colon data with reduced features having 9 genes, (B) heat map on lymphoma data with reduced features having 22 genes, (C) heat map of Leukemia data with reduced features having 14 genes

In Figure 3(Fig. 3), Performance of Proposed MOBPSO Algorithm, NSGA-II and GA on colon, lymphoma and leukemia datasets respectively using bayes Classifiers such as BLR, BN and NB are shown. In Figure 4(Fig. 4), Performance of Proposed MOBPSO Algorithm, NSGA-II and GA on colon, lymphoma and leukemia datasets respectively using Function based Classifiers such as LibLinear, LibSVM, Logistic, MLP, SGD and SPegasos are shown. In Figure 5(Fig. 5), Performance of Proposed MOBPSO Algorithm, NSGA-II and GA on colon, lymphoma and leukemia datasets using Tree based Classifiers such as BFT, DS, FT, J48, LMT, RF and REPT are shown. Figure 6(Fig. 6) demonstrates of heat maps for three datasets with reduced feature subsets of gene samples. The heat map is graphical representation of data to represent the level of expression of many genes across a number of comparable samples as they are obtained from DNA microarrays, where the individual values contained in a matrix are represented as colours. Larger values were represented by small dark gray or black colour and smaller values by lighter colours.

### Z-score analysis

Z scores provide a relative, semi quantitative estimation of microarray gene expression levels. Z score is calculated on the basis of hybridized intensity among experiments of same array type. Z score reflections on different hybridization values are as follows:

1. Z scores values with higher positive represent the genes with high expressiveness
2. Z scores values with Low negative values represent genes that are least expressed (Cheadle et al., 2003[3]).

Z scores are mathematically calculated as follows: 


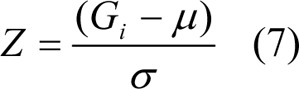


where G_i_ intensity of Gene, µ is mean of intensity G_1_...G_n_ (i.e. aggregate measure of all genes), and


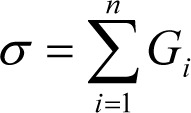


is SD.

**Figure 7:**
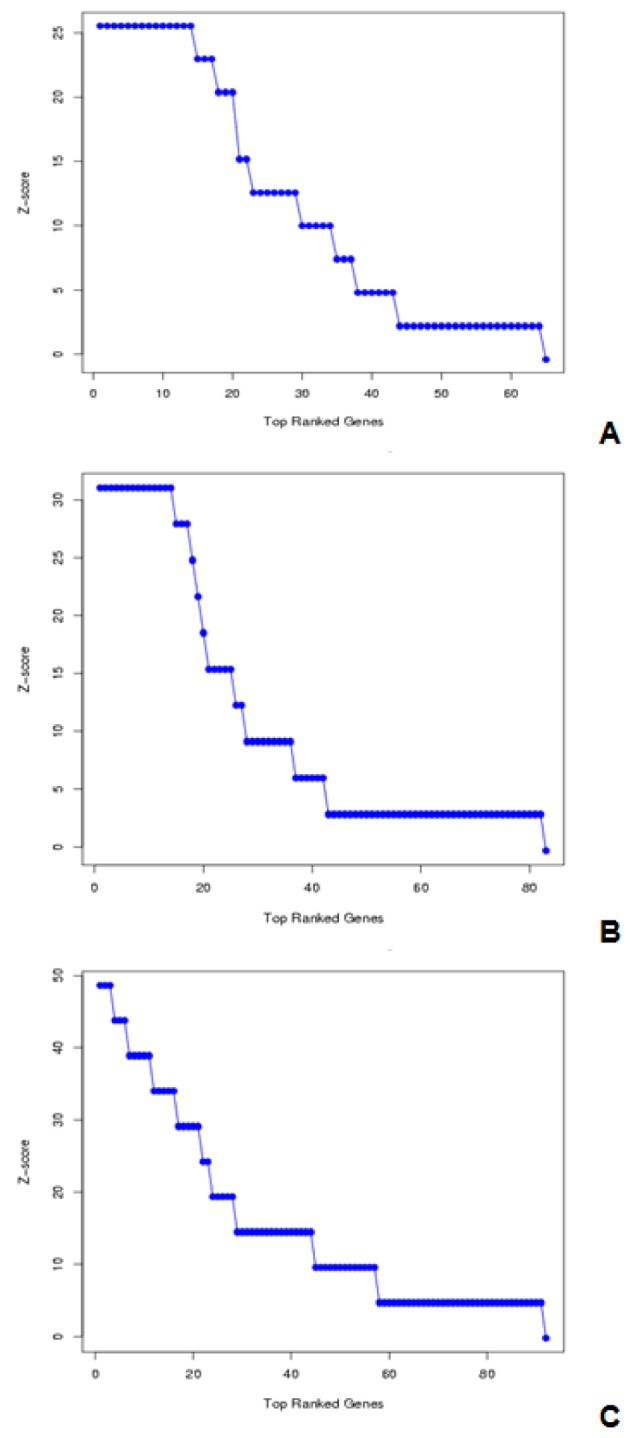
Z-Score Analysis of colon, lymphoma and leukemia having 9, 22, and 14 genes respectively; (A) Z-Score Analysis of colon having 9 genes, (B) Z-Score Analysis of Lymphoma having 22 genes, (C) Z-Score Analysis of Leukemia having 14 genes

In Figure 7(Fig. 7), Z score of colon, lymphoma and leukemia datasets, with a selected feature subsets and the selected genes, which are highly expressed to select are shown.

## Conclusion and Future Scope

In this paper, we presented a MOBPSO to find feature subset in cancer gene expression microarray data sets. Non-dominating sorting helps to preserve Pareto-font solutions. The pre-processing aids faster convergence along the search space and successfully employed to eliminate redundant and irrelevant features. The proposed approach is experimentally investigated with different parameters. The main goal of the feature selection is selecting minimal feature subsets with higher classification accuracy which has been achieved by two objective functions. The result on three benchmark cancer datasets demonstrates the feasibility and effectiveness of the proposed method. The performances of the proposed along with the existing methods are compared using standard classifiers and reported better and competitive performance.

## Conflict of interest

None declared.

## Figures and Tables

**Table 1:**
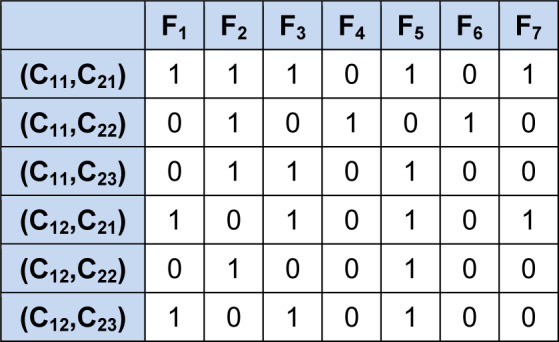
A simple example of a distinction table

**Table 2:**
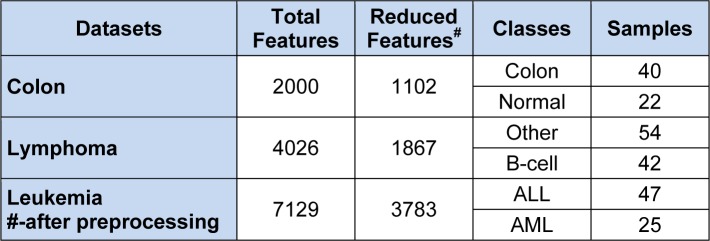
Details of the cancer microarray datasets before and after pre-processing

**Table 3:**
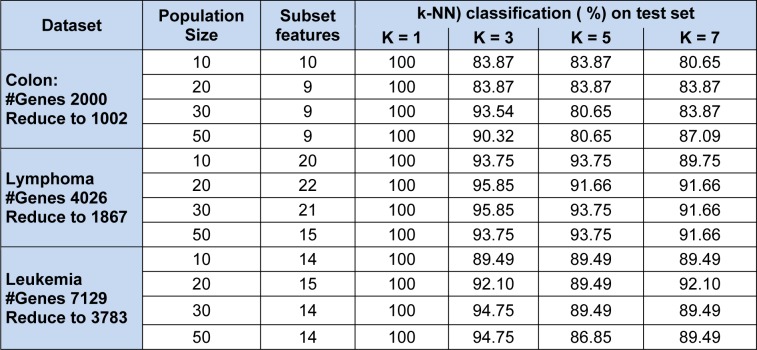
K-nearest neighbour (k-NN) classification results on colon microarray, lymphoma microarray, and leukemia microarray for the performance with proposed method

**Table 4:**
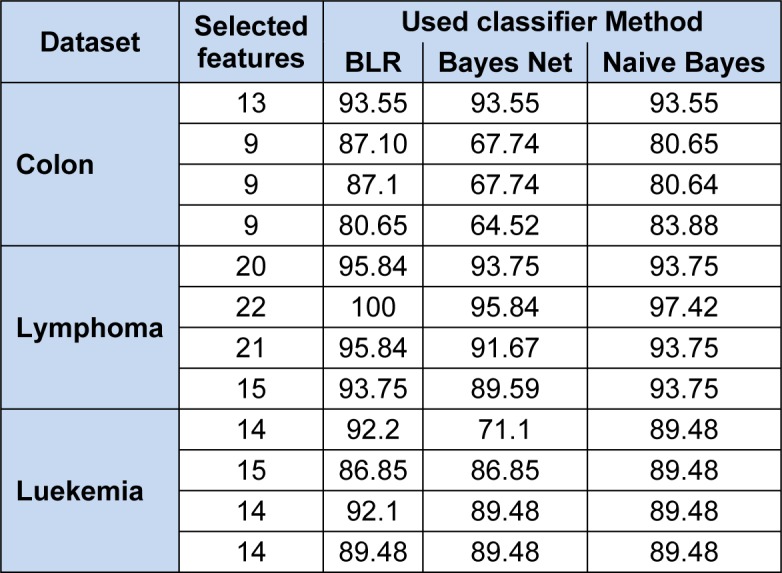
Performance on three datasets using Bayes family Classifiers

**Table 5:**
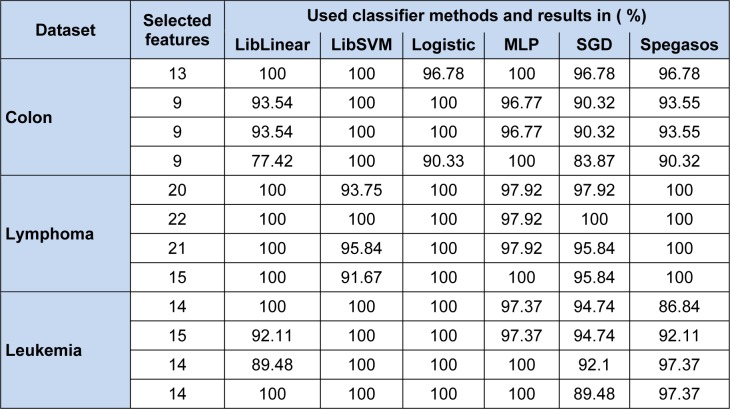
Performance on three datasets using Function Based Classifiers

**Table 6:**
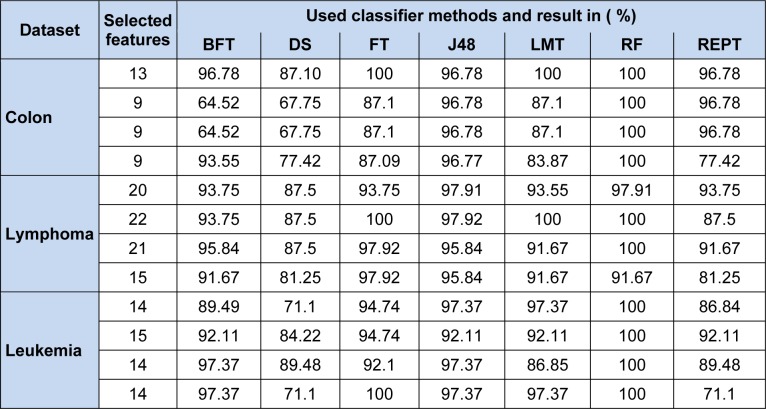
Performance on three datasets using Tree Based Classifiers

**Table 7:**
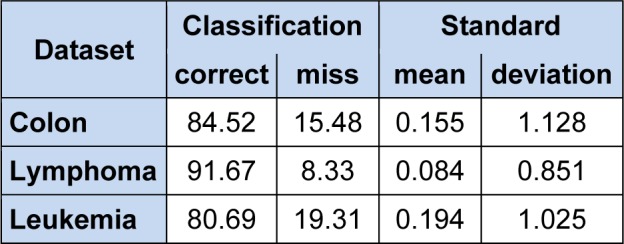
10-fold cross validation on colon, lymphoma and leukemia
